# A novel strategy to fuel cancer immunotherapy: targeting glucose metabolism to remodel the tumor microenvironment

**DOI:** 10.3389/fonc.2022.931104

**Published:** 2022-07-18

**Authors:** Xu Liu, Yujie Zhao, Xi Wu, Zhihui Liu, Xiaowei Liu

**Affiliations:** ^1^ Laboratory of Integrative Medicine, Clinical Research Center for Breast, State Key Laboratory of Biotherapy, West China Hospital, Sichuan University, Chengdu, China; ^2^ Department of Head, Neck and Mammary Gland Oncology, Cancer Center, West China Hospital, Sichuan University, Chengdu, China

**Keywords:** glucose metabolism, tumor microenvironment, aerobic glycolysis, immune checkpoint inhibitors, immunotherapy

## Abstract

The promising results of immunotherapy in tumors have changed the current treatment modality for cancer. However, the remarkable responses are limited to a minority of patients, which is due to immune suppression in the tumor microenvironment (TME). These include the pre-exists of suppressive immune cells, physical barriers to immune infiltration, antigen and antigen presentation deficiency, and expression of inhibitory immune checkpoint molecules. Recently, increasing evidence reveal that tumor metabolism, especially abnormal glucose metabolism of tumors, plays an essential role in tumor immune escape and is a potential target to combine with immunotherapy. By glucose uptake, tumor cells alter their metabolism to facilitate unregulated cellular proliferation and survival and regulate the expression of inhibitory immune checkpoint molecules. Meanwhile, glucose metabolism also regulates the activation, differentiation, and functions of immunocytes. In addition, tumor mainly utilizes glycolysis for energy generation and cellular proliferation, which cause the TME to deplete nutrients for infiltrating immune cells such as T cells and produce immunosuppressive metabolites. Thus, therapeutics that target glucose metabolism, such as inhibiting glycolytic activity, alleviating hypoxia, and targeting lactate, have shown promise as combination therapies for different types of cancer. In this review, we summarized the functions of glucose metabolism in the tumor cells, immune cells, and tumor microenvironment, as well as strategies to target glucose metabolism in combination with immune checkpoint blockade for tumor therapy.

## Introduction

During the past decade, multiple immunotherapies, such as immune checkpoint blockade, adoptive cell therapy, and cancer vaccines, have revolutionized the treatment of cancer (1–3). Notably, immune checkpoint inhibitors (ICIs) that restore the antitumor immune response of T-cells by blocking the ligation of coinhibitory signaling molecules (PD-1/PD-L1, CTLA4/B7) have become one of the most promising immunotherapies (4). Indeed, remarkable efficacy of ICIs has been shown in patients with several types of cancer (5, 6). However, most patients with cancer remain unresponsive to immunotherapy due to the tumor adopting multiple mechanisms to weaken the antitumor immune response. On one hand, tumor cells dampen immune responses through intrinsic mechanisms, such as loss of antigen presentation or expression of immunosuppressive molecules. On the other hand, the tumor microenvironment (TME), such as stromal barriers, hypoxia, insufficient vascularization, nutrient deficiencies, and metabolic disorders, also facilitates immunosuppression and limits anticancer immune responses.

Recently, the role of glucose metabolism in immune microenvironment has gained increased attention. Growing evidence reveals that glucose metabolism is able to aid tumor escape from immune surveillance and impede immunotherapy. Elevated glucose metabolism in tumor cells facilitates unregulated cellular proliferation and regulates the expression of inhibitory immune checkpoint molecules, such as PD-L1 (7). Meanwhile, glucose metabolism also regulates the activation and differentiation of immunocytes. It was reported that glycolysis could promote the function of T-cells and enhance the secretion of IFN-γ (8). Tumor-associated macrophage enhances glucose uptake and promotes differentiation of M1 macrophage, which increases the expression of pro-inflammatory cytokines and promotes tumor progression (9). Additionally, abnormal glucose metabolism also promotes the formation of the immunosuppressive microenvironment. Tumors mainly utilize glycolysis for energy generation, which causes depletion of nutrients for infiltrating immune cells and production of immunosuppressive metabolites (8, 10). Moreover, high glucose metabolism transforms TME into acidic and hypoxic phenotypes, which dampen the cytotoxicity activity of T-cells (11–13). Indeed, targeting tumor cell glycolysis not only inhibits tumor cell growth, but also preserves the antitumor T-cell function (14).

In conclusion, aberrant glucose metabolism in tumors may lead to immune cell dysfunction, which may account for the failure of immunotherapy. An in-depth understanding of tumor immunometabolism may contribute to discovering novel promising approaches to boost T-cells activity. Here, we reviewed the characteristics of glucose metabolism in tumor microenvironment, discussed the regulatory role of glucose metabolism in antitumor immune response, and summarized the potential therapeutic strategies targeting glucose metabolism to enhance the efficacy of cancer immunotherapy, especially ICIs.

## Characteristics of glucose metabolism in the tumor microenvironment

Highly proliferative cancer cells uptake large amounts of glucose for energy generation in the TME. Likewise, the differentiation and activation of immunocytes in the TME also rely on glucose metabolism, thus creating a competition for glucose between cancer and immune cells (15). The intensive glycolysis of tumor cells impedes T-cell access to glucose, thereby impairing their appropriate proliferation and function (16). Dysregulation of glucose metabolism in the TME can affect the biological activity of tumor cells and also regulate the effector functions of immune cells. Therefore, a better understanding the changes of glucose metabolism in the TME and their impacts on antitumor immune response may help uncover potential metabolic targets to inhibit tumor growth and enhance the efficacy of immunotherapy.

### Glucose metabolism of tumor cells

Notably, the level of glycolysis is increased in tumors despite sufficient oxygen levels. This phenomenon of metabolic reprogramming, also known as the Warburg effect, is considered a crucial feature of cancer metabolism (17, 18). Although oxidative phosphorylation (OXPHOS) is a more efficient pathway for ATP generation, tumor cells have a lower rate of OXPHOS (18). Apparently, glycolysis is an important metabolic pathway for highly proliferative cancer cells, which can supply essential metabolic intermediates to synthesize biomolecules, such as nucleotides, lipids, and amino acids (19). The programs of glucose metabolism of tumor cells consume lots of glucose, then convert glucose into pyruvate and generate ATP. Further metabolic reactions of pyruvate release lactate into the TME. Activation of PI3K/AKT signaling in tumor cells increases glucose transporters (GLUTs) at the plasma membrane and promotes glucose uptake. AKT has also been found to activate the glycolytic enzymes (20). Upregulation of glycolytic metabolism in tumors results in glucose depletion, hypoxia, and lactate accumulation in the TME. In return, hypoxia further promotes glycolysis in tumor cells by stabilizing hypoxia-inducible factor-1α (HIF-1α), which induces the transcription of glycolytic enzymes (21). The high levels of lactate result in acidic conditions that promote tumor progression and metastasis (22, 23). However, glucose depletion and hypoxia impose metabolic stress on T-cells and impair the cytolytic activity (16). In addition, lactate has diverse effects on immune cells, including polarizing macrophages towards the anti-inflammatory M2 type, supporting regulatory T-cells or suppressive myeloid populations, and interfering with dendritic cell (DC) maturation (24, 25). Collectively, these findings indicate that glycolytic activity not only provides advantages to cancer cells to grow under hypoxia and glucose deprivation conditions but also creates an immunosuppressive microenvironment to regulate surrounding cells that contributes to tumor immune evasion. Therefore, targeting glucose metabolism to improve the TME is a rational and promising strategy for anticancer therapy.

### Glucose metabolism of immune cells in tumor

Importantly, metabolic reprogramming also occurs within immune cells (26). T-cells are critical executors in tumor cell killing. The reprogramming of glucose metabolism is involved in the differentiation and activation of T-cells. Briefly, naïve CD4^+^ and CD8^+^ T-cells have minimal metabolic activity and engage in OXPHOS to generate energy. Upon activation, the activated T-cells are accompanied by metabolic changes that increase glucose uptake and upregulate glycolysis to support their growth and function (27). The glucose metabolism of activated T-cells is regulated by several signaling pathways, including the PI3K/AKT/mTOR and AMPK pathways (28, 29). The PI3K/AKT/mTOR pathway senses nutrient availability and promotes glucose uptake and glycolysis (30), whereas AMPK induces a metabolic switch toward OXPHOS by inhibiting mTOR signaling (31, 32). Hence, glucose depletion within the TME enhances AMPK activation, which may inhibit effector function of T-cells (33). Nutrient competition between cancer and immune cells contributes to functional exhaustion of CD8^+^ T-cells (34, 35). Exhausted T-cells also exhibit dysregulated metabolism with repressed glycolytic and mitochondrial function (36, 37). In addition, CD8^+^ memory T-cells are an important member of the adaptive immune system and play a crucial role in long-term tumor control. During the differentiation of activated effector T-cells into memory T-cells, there is also a metabolic shift to increase mitochondrial metabolism for energy production (38). Inhibition of mTOR signaling enhances CD8^+^ memory T cell formation but compromises effector T-cell activity (39). Antitumor CD4^+^ T-cells share metabolic profiles with activated CD8^+^ T-cells (40). However, not all CD4^+^ T-cells undergo metabolic transition after activation. Regulatory T-cells (Treg), a subset of CD4^+^ T-cells, play a critical role in dampening antitumor immune response. Low-glucose availability induces FOXP3 expression, increasing T cell differentiation to Tregs (39). This immunosuppressive subset has low levels of glycolysis and predominantly depends on mitochondrial respiration and fatty acid oxidation for their metabolism (41, 42). In addition, lactate is preferentially utilized by Tregs since they prefer oxidative metabolism (42). The distinct metabolic programs enable Treg cells to maintain optimal function in the lactate-rich TME (42).

The activation of natural killer (NK) cells depends on glycolysis and OXPHOS to supply energy (43). Therefore, the lack of glucose in the TME affects the function of NK cells. It has been reported that the glycolytic rate of NK cells was suppressed in lung cancer, which further weakened the cytotoxicity and cytokine production (44). Glycolysis is not the only way of glucose metabolism, gluconeogenesis also plays a significant role in TME (45). Gluconeogenesis is the process of generating glucose from non-carbohydrate substrates. Fructose-1,6-bisphosphatase (FBP1), a key enzyme in gluconeogenesis, is upregulated in murine NK cells (44). FBP1 represses the expression of glycolytic genes and reverses the direction of glycolysis to promote glucose synthesis (46). Therefore, inhibition of FBP1 strongly restores glycolytic metabolism and improve the function of NK cells (44).

Similarly, other innate immune cells employ specific metabolic programs upon activation. DCs are professional antigen presenting cells that phagocytize and present antigen to T-cells after maturation. This process is accompanied by an increase in glycolysis through PI3K/AKT pathway in response to Toll-like receptor (TLR) signals (47). Activated DCs require high glycolytic metabolism, which is critical for DC continued survival and secretion of pro-inflammatory cytokines (48). Thus, glucose competition in the TME may affect DC function and limit the activity of T-cells (49). Macrophages reprogram their metabolism in the TME through elevated glycolysis while maintaining a tumor-promoting function (50). Tumor-associate macrophages (TAMs) are categorized into inflammatory (M1) and immunosuppressive (M2) phenotypes, which have distinct metabolic programs. The pro-inflammatory M1 macrophages enhance glycolysis and fatty acid synthesis (FAS) to support their function (51). Conversely, like Treg cells, anti-inflammatory M2 macrophages rely more on fatty acid oxidation (FAO) and OXPHOS to maintain their tumor-promoting function (50, 52). The myeloid-derived suppressor cells (MDSCs) also exhibit characteristic metabolic phenotypes. It is generally believed that both aerobic glycolysis and OXPHOS were upregulated in MDSCs (53). Recent study shows that MDSCs consume the most glucose per cell in the TME and account for one-third of glucose uptake in the whole tumor, limiting the availability of glucose to other immune cells (54). And blocking glycolysis has been shown to inhibit the expansion and function of MDSCs (55). Hypoxia and lactate in the TME can polarize macrophages toward M2 anti-inflammatory phenotype, promote MDSC suppressive function, and reduce dendritic cell activation and stimulatory effects (56–58).

## Targeting glucose metabolism to enhance cancer immunotherapy

Multiple researches have shown that glycolytic activity of glucose metabolism not only provides an intrinsic growth advantage for cancer cells but also exerts inhibitory effects on antitumor immune cells by creating an immunosuppressive microenvironment. Thus, improving the immune state of TME *via* metabolic intervention is expected to provide promising strategies for enhancing the therapeutic effect of tumors. To date, several drugs have been proposed to target tumor glucose metabolism for cancer treatment (**Table 1**). Here, we summarize the therapeutic strategies targeting glucose metabolism and advances in glucose metabolism intervention combined with ICI immunotherapy.

**Table 1:** Potential targets for modulating glucose metabolism.

Intervention	Target	Representative drugs
Inhibiting glycolytic activity	HK	2-DG
	PFK-1	DCA
	GLUT1	WZB117
Targeting lactate in the TME	LDH-A	FX11, Galloflavin
	MCTs	Lenalidomide, AZD3965
	Acidic pH	Bicarbonate
	Proton pump	Omeprazole
Alleviating hypoxia in the TME	Hypoxia	Supplemental oxygen, Evofosfamide
	Mitochondrial respiratory complex I	Metformin
Targeting PI3K/AKT/mTOR signaling pathway	mTOR	Rapamycin
	PI3K isoforms	AZD8835, BKM120
	AKT	AKTi, Ipatasertib

HK, Hexokinase; 2-DG, 2-Deoxyglucose; PFK-1, Phosphofructokinase-1; DCA, Dichloroacetate; GLUT1, glucose transporter 1; LDH-A, Lactate dehydrogenase-A; MCTs, Monocarboxylate transporters; mTOR, mammalian target of rapamycin; PI3K, phosphatidylinositol-3 kinase.

### Strategies of targeting glucose metabolism within the TME

#### Inhibiting glycolytic activity of tumor cells

The enhanced glycolytic activity of cancer cells results in deprivation of glucose and accumulation of lactate, which inhibit the effector function of antitumor immune cells and promote the differentiation and recruitment of immunosuppressive cell populations. Therefore, weakening tumor aerobic glycolysis by either inhibiting glycolysis-related enzymes or using the competitive glucose analog may help to inhibit tumor growth. 2-Deoxyglucose (2-DG) is a glucose analog and inhibitor of hexokinase used to reduce glycolysis. Studies revealed it could inhibit cancer cell proliferation and promote the formation of memory CD8^+^ T-cells, but it also impairs the effector function of T-cells (59, 60). Dichloroacetate (DCA) is an inhibitor of phosphofructokinase-1 and can induce a shift from glycolysis to OXPHOS and inhibits tumor cells’ growth (61, 62). However, these two drugs also inhibit T-cell function and promote immunosuppression because they are not specific to tumor cell metabolism. Therefore, the targeting specificity of tumor cells is critical for applying glycolysis inhibitors.

#### Targeting lactate in the TME

The enhanced glycolytic activity of tumor cells can release large amounts of lactate, leading to the formation of acidic TME (63). Lactic acid increases the expression of IL-8 and VEGF, which promotes tumor metastasis and progression (22). Yet, lactate inhibits the proliferation and cytotoxicity of T and NK cells, but promotes the survival of Treg and MDSC cell populations (42, 64). Hence, inhibiting the production of lactate or neutralizing lactate in the TME may improve the efficacy of immunotherapy. Lactate dehydrogenase (LDH), which is comprised of two major subunits, LDH-A and LDH-B, catalyzes the reversible reaction between pyruvate and lactate. LDH-A has a higher affinity for pyruvate and favors the conversion of pyruvate to lactate, making LDH-A a target of interest (65). LDH-A inhibitors, such as FX11 and galloflavin, have been reported to reduce tumor growth (66). Another approach to targeting lactate is to inhibit lactate transporters monocarboxylate transporter (MCT) 1-4. MCT inhibitor lenalidomide has been reported to suppress tumor cell proliferation and enhance IL-2 and IFN-γ secretion of T-cells (67). In a mouse model of breast cancer, blockade of MCT1/MCT4 combined with ICI enhances antitumor immune responses compared to ICI alone (14). Further, neutralizing lactic acid with bicarbonate or proton pump inhibitors can improve the low pH of TME. Importantly, oral bicarbonate combined with anti-PD-1 immunotherapy inhibits tumor growth in a melanoma mouse model (68, 69).

#### Alleviating hypoxia in the TME

The rapid proliferation of tumor cells results in massive oxygen consumption and hypoxia in the TME. Under hypoxic conditions, HIFs are stabilized to promote the expression of glycolytic genes and cytokines that promote tumor metabolism and angiogenesis (70). Furthermore, intra-tumoral hypoxia inhibits the function of T-cells and supports the generation of immunosuppressive cells, such as Treg and M2 macrophages (71, 72). As oxygen is a determinant of energy production needed for the differential and activation of effector T-cells, alleviating hypoxia can boost antitumor immune responses. Studies have confirmed that supplemental oxygen increased T-cell infiltration and inflammatory cytokine production, and decreased Treg cell, which can enhance the antitumor immunity of T-cells in mice with lung tumors (73). Similar results can be obtained with the use of metformin, which reduces tumor hypoxia and improves T-cell response in a mouse model of melanoma (89).

#### Targeting PI3K/AKT/mTOR signaling pathway

The PI3K/AKT/mTOR is an important pathway involved in cell proliferation. This pathway also senses nutrients and plays a key role in promoting glycolysis in tumor cells and effector T-cells (39). Analogs of rapamycin can reduce cancer cell aerobic glycolysis and proliferation. However, these drugs can also suppress the function of effector T-cells and endow T-cell with a memory phenotype (74). For example, the mTORC1 inhibitor rapamycin enhances the formation of CD8^+^ memory T-cells and also inhibits immune response of CD8^+^ T-cells (75). Likewise, PI3K inhibitor skews T-cell differentiation toward memory phenotypes and improves *in vivo* persistence and antitumor activity in mice with acute myeloid leukemia (76). Among the PI3K isoforms, PI3Kα and PI3Kβ are ubiquitously expressed, whereas PI3Kγ and PI3Kδ are found primarily in leukocytes. PI3Kα/δ inhibition promotes antitumor immunity through enhancement of CD8^+^ T-cell activity and suppression of Tregs (77). Interestingly, recent evidence suggests that rapamycin combined with immunotherapy promote cytotoxic and memory function of T-cells in glioblastoma (78). Therefore, blockade of PI3K/AKT/mTOR signaling may be an attractive strategy to be used in combination with immunotherapy.

### Advances in targeting glucose metabolism combined with immune checkpoint inhibitors

Immune checkpoint inhibitors restore the antitumor immunity of T-cells by blocking the binding of inhibitory immune checkpoint ligands and receptors. Undoubtedly, ICIs have achieved good results in some malignant tumors. Recent evidence has indicated that immune checkpoint signaling can regulate metabolism of cancer cells and T-cells. Indeed, several studies suggest that the interaction of PD-1 and PD-L1 impairs the metabolic feature of T-cells, including inhibiting aerobic glycolysis *via* suppression of PI3K/AKT/mTOR signaling (79, 80). Similar to PD-1signaling, CTLA-4 ligation to CD80/CD86 inhibits the glucose metabolism of T-cells by reducing AKT phosphorylation and activation (81). Hence, checkpoints signaling would impact the activation and antitumor function of T-cells (80). In addition, immune checkpoints also affect metabolism of tumor cells. PD-L1 in tumor cells has been shown to upregulate aerobic glycolysis by increasing the activity of PI3K/AKT/mTOR pathway (82). Consequently, PD-1/PD-L1 axis supports the survival and progression of cancer cells. Thus, inhibition of PD-1/PD-L1 signaling might restore the metabolic requirements of T-cells while simultaneously inhibiting glycolysis levels in tumor cells. Indeed, the ICIs differentially influence the metabolic programs in the TME by increasing the glucose availability to T-cells and in contrast inhibiting the rate of glycolysis in cancer cells. In tumors, the TME imposes many metabolic stresses on immune cells, especially the antitumor effector T-cells. Therefore, the metabolic treatments that modulate glucose metabolism to improve the TME could be attractive adjuvants to be used in combination with ICIs.

Recently, researchers constructed a nanosystem embedded with lactate oxidase (LOX) and a glycolysis inhibitor for lactate consumption and modulation of metabolism. This nanosystem can inhibit tumor growth by blocking glycolysis and removing lactic acid in the TME. Importantly, this metabolic regulation strategy can effectively improve the therapeutic effect of anti-PD-L1 therapy and avoid systemic toxicity (83). In addition, other studies also found that neutralizing tumor acidity with oral bicarbonate impaired the tumor growth in melanoma mice, further combining bicarbonate with anti-PD1 improved antitumor response and prolonged mouse survival (68). Metformin can inhibit oxygen consumption in tumor cells to reduce intra-tumoral hypoxia. A combination of metformin with PD-1 antibody improves T-cell function and tumor clearance in mice with melanoma (84). Favorable treatment outcomes were also observed in melanoma patients receiving metformin in combination with ICIs (85).Similarly, patients with non-small cell lung cancer receiving concurrent metformin and ICIs showed higher response rate and overall survival (86). A Phase I clinical trial reported that evofosfamide in combination with ipilimumab improved immune activity in advanced solid tumors (87). Future studies focusing on metabolic modulation therapy in combination with ICIs are clearly warranted.

## Conclusion and perspectives

Increasing evidence indicates that cellular metabolism could remodel TME and regulate antitumor immunity. These provide opportunities to target metabolism as a means to enhance immunotherapy. Varieties of strategies targeting glucose metabolism have been developed for tumor therapy, but their efficacy needs to be further explored. Moreover, the similarity of metabolism between tumor cells and T-cells raises the concern that targeting tumor metabolism might undermine the immune response of activated T-cells. In fact, several studies have suggested that drugs targeting tumor aerobic glycolysis also significantly inhibit T-cell function and promote immunosuppression (59). Therefore, special attention should be devoted to targeting tumor cells and avoiding systemic toxicity. Using more specific inhibitors or nano-delivery technology may be the way to address the toxic effects.

In addition to glucose metabolism, tumor cells also obtain energy and substances through other metabolic pathways, such as amino acid and fatty acid metabolism. Hence, it is unlikely that a single enzyme or transporter targeting specific pathway will provide a perfect solution. Instead, approaches intervening metabolic targets in combination with other therapies, including ICIs, may offer the most significant potential to improve clinical efficacy.

## Author contributions

All authors listed have made a substantial, direct, and intellectual contribution to the work and approved it for publication.

## Funding

This work was funded by National Natural Science Foundation of China (No. 22105137 and 81773752), China Postdoctoral Science Foundation (No. 2020M683324), Post-Doctor Research Project, West China Hospital, Sichuan University (No. 2020HXBH051), Key Program of the Science and Technology Bureau of Sichuan (No. 2021YFSY0007).

## Conflict of Interest

The authors declare that the research was conducted in the absence of any commercial or financial relationships that could be construed as a potential conflict of interest.

## Publisher’s note

All claims expressed in this article are solely those of the authors and do not necessarily represent those of their affiliated organizations, or those of the publisher, the editors and the reviewers. Any product that may be evaluated in this article, or claim that may be made by its manufacturer, is not guaranteed or endorsed by the publisher.
